# Vaccination Status and Associated Factors among Street Children 9–24 Months Old in Sidama Region, Ethiopia

**DOI:** 10.5334/aogh.2650

**Published:** 2020-01-06

**Authors:** Ababe Tamirat Deressa, Melesse Siyoum Desta, Teshome Melesse Belihu

**Affiliations:** 1 School of Nursing, College of Medicine and Health Sciences, Hawassa University, ET; 2 Department of Midwifery, College of Medicine and Health Sciences, Hawassa University, ET

## Abstract

**Background:**

Childhood non-vaccination can have different short-and long-term negative outcomes on their health. In Ethiopia, in addition to low coverage of full vaccination, street children were among the neglected part of the community who were missed during planning and reporting vaccination coverage. Moreover, there is no related research conducted on this title specifically.

**Objective:**

The objective of the study was to assess the vaccination status and its associated factors among street children 9–24 months old in Sidama zone.

**Methods:**

Community-based cross-sectional study design was conducted in four selected towns of Sidama region, southern Ethiopia. The convenience sampling method was applied to involve mothers of street children younger than two years during the study period. Data entry was done with EpiData version 3.1 and exported to SPSS22 for analysis. Bivariate and multivariable logistic regression analysis were performed to identify factors associated with immunization status of street children.

**Results:**

A significant number (26 [24.3%]) of the street children younger than two years were not vaccinated. Those mothers who are ≤20 years old (P = 0.014, AOR = 0.216, 95% CI: 0.064–0.732) and who gave birth at home (P = 0.029, AOR = 0.292, 95% CI: 0.097–0.879) had less odds of vaccinating their child than those older than 20 and who gave birth at health facility respectively.

**Conclusion:**

A significant number of the street children in this study are not fully vaccinated. Mothers aged <20 years and home births were significantly associated with non-vaccination status.

## Background

Vaccination is defined as “the artificial induction of active immunity by introducing host the specific antigen (living modified agent, killed organism, or inactivated toxin) of a pathogenic organism into a vulnerable [1].” The Expanded Program on Immunization (EPI) was established by World Health Organization (WHO) established in 1974. Firstly, the program aimed immunization of children against six vaccine-preventable diseases (VPDs) i.e. polio-myelitis, neonatal tetanus, measles, diphtheria, pertussis, and tuberculosis [2].

As it is indicated from global effect of maternal education on childhood vaccination, only 42.8% of mothers who had no education fully vaccinate their children. This almost gets doubled (80.2%) amongst children whose mothers had completed tertiary education [3].

Childhood non-vaccination can have different short-and long-term negative outcomes on their health. For instance, among unimmunized children living in slums of India, it was evidenced as malnutrition is their common problem [4].

In Southern Nepal, the largest share (76%) of infants had no OPV vaccination by four months while only 8% have been fully vaccinated by the age of six months. Even though it is recommended at birth, only half (49%) of children had received a BCG vaccine by the age of months old [5].

The qualitative report from Nigeria stated that there is misconception towards immunization. For instance an individual from the rural side said, “Immunization is given when malaria wants to set in. If a child receives the injection, the malaria will no longer manifest. Also even if the malaria has occurred, immunization will make it not to be too much [6].” During 2007 in rural Mozambique, only 21.2% of mothers had heard about the EPI as a specific program [7].

The proportion of fully vaccinated children in Ethiopia increased by 15%, from 24% in 2011 to 39% in 2016 [8]. In rural Ethiopia, children living ≥60 minutes from a health post were found to receive Penta3 vaccine compared to children living <30 minutes from a health post. However, no association was realized between vaccination coverage and wealth status of the household [9].

There was less (<20%) coverage of routine vaccinations for children aged 12–23 months in both Ambo and Yaya-Gulelena D/Libanos district, Ethiopia [10]. A significant number (40.7%) of children in Ambo woreda, Ethiopia, received one or more vaccines but did not complete all the recommended doses, and only 35.6% of them completed all the recommended vaccines. More than three quarters (77%) of respondents were aware of less than three types of vaccines for preventable diseases and only 23.3% of them mentioned four or more types of vaccines for preventable diseases [11].

Even though the UN is paying attention to the pledge of “Leaving no one behind [12],” street children are still ignored part of the community in different parts of the world. Also, the magnitude of the problem of street children has not been fully studied in Ethiopia. In line with this, research has not touched the issue of non-vaccination among street children in the current study setting. Thus, this study aimed to identify the vaccination status among street children and its associated factors so as to contribute for improvement of immunization coverage among street children.

## Methods

### Aim of the Study

The study aimed to assess vaccination status and associated factors among street children 9–24 months old in Sidama region, southern Ethiopia, in 2019.

### Study Design and Sampling

Community-based cross-sectional study design was conducted. Since there is no identified number of street children younger than two years in the area, though they are believed to be an addressable size, all street mothers with children 9–24 months old were surveyed. Towns/cities that are considered to have street children from the region (Hawassa, Yirgalem, Aleta wondo, and Bensa) were purposively selected. The convenience sampling method was applied to involve all available mothers of street children 9–24 months old in the zone during the study period. Data were collected using a structured questionnaire that was developed from review of different literatures.

### Data Management and Analysis

A data collection tool was initially developed in English and translated to Amharic and Sidamigna (the local language) then back to English. A pretest was conducted in Shashemene town. Data were collected by trained nurses and midwives. Data coding and entry were accomplished using EpiData3.1 and exported to SPSS 22. Data cleaning, recoding, and analysis were performed with this SPSS. Bivariate and multivariable logistic regression analysis were done to test association between dependent and independent variables. After checking associations of the variables, those with p < 0.2 in bivariate analysis were processed to multivariable logistic regression analysis to control confounding factors in the association. Finally, a P-value of <0.05 was considered to indicate statistical significance.

## Results

A total of 107 street mothers with children of under two years were involved in the study.

### Socio-demographic Characteristics

The minimum and maximum age of mothers were 15 and 40 years old respectively with a mean maternal age of 26.69 (+5.269). A majority (84.1%) of them were older than 20 years. Sixty-four mothers in this study (59.8%) were married and 56 (52.3%) follow Protestant religion. Almost three quarters (74.8%) of them were street off mothers (Table 1). The mean age of the children was 18.33 (+5.485) months. The largest share (51.4%) of the children are male and female accounts for 48.6% (52).

**Table 1:** Socio-demographic characteristics of the street children’s mothers, April, 2019 (n = 107).

Variables	Frequency		Percent

**Age**	≤20	17	15.9
	≥21	80	84.1
**Marital status**	Unmarried	14	13.1
	Married	64	59.8
	Widow	12	11.2
	Divorced	16	15
	Cohabited	1	0.9
**Religion**	Protestant	56	52.3
	Orthodox	42	39.3
	Muslim	4	3.7
	Catholic	1	0.9
	No religion	4	3.7
**Ethnicity**	Sidama	66	61.7
	Wolaita	17	15.9
	Oromo	7	6.5
	Amhara	10	9.3
	Others*	7	6.5
**Education status**	No formal education	53	49.4
	Primary school	50	46.7
	Secondary school	4	3.7
**Character of dwelling**	Street on	27	25.2
	Street off	80	74.8

* Gedeo, Guraghe, Hadiya, Kembata and Tigre.

### Obstetric Factors

**Figure 1:**
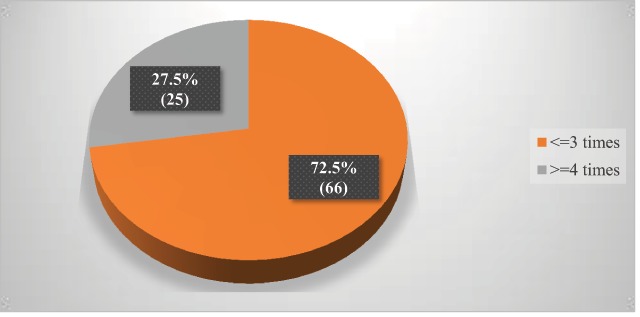
Frequency of ANC follow up during pregnancy of the index child, April, 2019 (n = 91).

From a total of 107 surveyed mothers, 89.7% (96) reported as they have ≤2 children under five and 10.3% (11) of them had ≥3 children under five. Ninety-one (85%) of the mothers had ANC follow-up during pregnancy of the index child and 15% (16) had no ANC follow up. A high percentage of them had follow-ups ≥4 times (Figure 1).

Most of the mothers (77.6%) gave birth to the index child at a health facility and 22.4% (24) of them reported that they gave birth at home (where they live).

### Mother’s Knowledge about Vaccination

**Figure 2:**
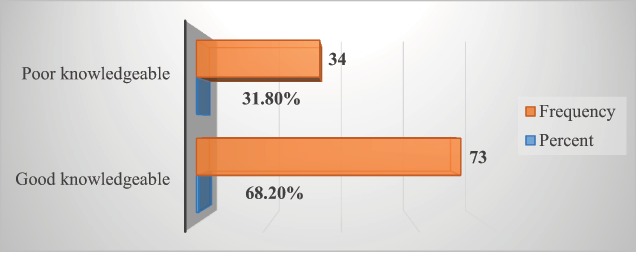
Mothers’ level of knowledge towards vaccination, April, 2019 (n = 107).

In this study, 31.8% (34) of them have poor knowledge about vaccination (Figure 2). Almost all (98.1%) mothers have heard about vaccination from various sources. From these, 88.6% (93) heard from health professionals and others from advertisements on the street (5.7%), radio (1.9%), television (1.0%), and other sources of information (2.9%) including friends and neighbors. Seventy-three (69.5%) of the mothers aware that vaccination is available at any public health facility and others (30.5%) are not. From those who have heard about vaccination, 93.3% (98) considered that vaccination is a free service at a public health facility. In addition, 95.3% (102) of street mothers in this study reported that vaccination can prevent disease and a few (4.7%) didn’t believe vaccination prevents disease.

Except for a few (1.9%), the majority (98.1%) of the mothers in this study reported that they know the nearest public health facility to their dwelling site during data collection. A smaller number (3.8%) travel more than 30 minutes to reach the nearest public health facility, while 96.2% (101) travel less than or equal to 30 minutes to reach the nearest public health facility. A majority (60.7%) responded that the right time to initiate vaccination is birth to two weeks after birth and 2.8% (3) of them don’t know the right time to initiate vaccination (Table 2).

**Table 2:** Responses of the street children’s mothers to the right time to initiate vaccination, April, 2019 (n = 107).

Responded time of initiation	Frequency	Percent

Birth to 2 weeks	65	60.7
2–4 weeks after birth	24	22.4
4–6 weeks after birth	10	9.3
6 weeks after birth	1	.9
Any time within one year of birth	4	3.7
Don’t know	3	2.8

### Vaccination Status

In this study, 81 (75.7%) of the mothers reported that their 9–24 month old children had been fully vaccinated. The largest share (83.5%) of the children were vaccinated for Penta 1. The percentage of children vaccinated for Penta 2 and Penta 3 was 81.6%, whereas those who had been vaccinated for BCG and measles was 72.8%.

### Factors Associated with Vaccination Status

In bivariate analysis, place of delivery, maternal age, character of dwelling, and ANC follow-up during pregnancy of the child showed significant association with the vaccination status of the child younger than two years. However, character of dwelling and ANC follow-up during pregnancy was insignificant in multivariable logistic regression analysis.

Those mothers who gave birth at home were 70.8% less likely to vaccinate their child than those who gave birth at a health facility (P = 0.029, AOR = 0.292, 95% CI: 0.097–0.879). On the other hand, mothers 20 years old or younger were 78.4% less likely to vaccinate their child than those older than 20 years (P = 0.014, AOR = 0.216, 95% CI: 0.064–0.732) (Table 3).

**Table 3:** Factors associated with vaccination status among street children 9–24 months old in Sidama zone, April, 2019.

Variables		Vaccinated		COR	AOR (CI)	P-value

		No	Yes			

Place of delivery for the index child	Home	10 (41.7%)	14 (58.3%)	0.334	**0.292 (0.097–0.879)**	0.029**
	Health facility*	16 (19.3%)	67 (80.7%)	1	1	
Maternal age	≤20	9 (52.9%)	8 (47.1%)	0.207	**0.216 (0.064–0.732)**	0.014**
	>20*	17 (18.9%)	73 (81.1%)	1	1	
Dwelling character	Street on	11 (40.7%)	16 (59.3%)	0.336	0.489 (0.172–1.39)	0.179
	Street off*	15 (18.8%)	65 (81.3%)	1	1	
ANC follow-up	Yes	18 (19.8%)	73 (80.2%)	4.056	1.56 (0.435–5.59)	0.495
	No*	8 (50%)	8 (50%)			

* Reference category, ** statistically significant association.

## Discussion

The magnitude of fully vaccinated children in this study is higher than the report from both Ambo and Yaya-Gulelena Ad/Libanos district, Ethiopia [11]. Again, the magnitude is higher than report of vaccinated children from Jigjiga, Eastern Ethiopia [13]. The possible reason for this variation may be the study setting, data collection approach and year variation in study period. In this study, mother’s response was used to identify whether the child was vaccinated or not, and this may increase the proportion of vaccinated children. However, the reports of incomplete vaccination schedules is comparable with that of the Ambo and Yaya-Gulelena Ad/Libanos district, Ethiopia [11]. On the other hand, the proportion of fully vaccinated children in this study is lower than the report from Sinana district, southeast Ethiopia [14], 2016 EDHS [8], India [15], Bangladesh DHS [16] and Umraniye, a district of Istanbul, Turkey [17]. The variation may be due to a difference in study participants’ characteristics. All study participants in this study were street mothers who might be less concerned about immunizing their children as they may be burdened by other aspects of survival, such as begging.

The proportion of mothers who had ANC follow-up during pregnancy (85%) is higher than the national report of 2016 EDHS [8], which was 62%. All street mothers in this study are from towns where they can easily access a health facility for ANC service and this could be the reason for the variation because a national report includes rural mothers where health facilities may not be easily accessed. The other reason may be the time variation; this study was conducted three years after that of the national report.

Almost all mothers of the street children in this study have heard about vaccination. This is much more than the percentage of mothers who heard about vaccination in rural Mozambique (21.2%). The current study was conducted in an urban setting where there is more exposure to information about vaccination than in the rural setting. This might be the reason for this significant variation [7].

A significant number of street mothers in this study had poor knowledge about vaccination and didn’t know the right time to initiate vaccination. Educated persons search and analyse information from different sources to learn about different issues, so this may be due to the low educational status of the mothers in this study; a majority of them had no formal education or attended primary school. This is comparable with the study report from Egypt [18].

The coverage of Penta 1 vaccination in this study was higher than that of BCG, Penta 2 and 3 and measles. This may be due to the mothers initiating vaccination at an inappropriate time and quitting the late schedules of immunization as a significant number of mothers did not know the right time to initiate immunization.

The proportion of the mothers in this study who reported that vaccine can prevent disease is comparable with the proportion of mothers (83%) who reported that vaccine is effective in the study findings of Mysore, India [19].

Distance from health facility in this study had no significant association with vaccination status, unlike another study in rural Ethiopia [9]. The possible reason for this difference might be the variation in study setting; participants in this study were mothers in an urban region where they were able to find a health facility nearby.

Those mothers who gave birth at home were 0.708 times less likely to vaccinate their child than those who gave birth at a health facility (P = 0.029, AOR = 0.292, 95% CI: 0.097–0.879). It is possible that those mothers who gave birth at a health facility might have been educated about vaccination in a health institution. Additionally, if it is an institutional delivery, by default they also receive OPV0 and BCG.

On the other hand, those mothers younger than 20 years had lower odds (P = 0.014, AOR = 0.216, 95% CI: 0.064–0.732) of vaccinating their children than their counterparts. The possible reason behind of this might be experiences from a previous birth and even learning from their previous mistakes, such as not vaccinating the child, as those mothers older than 20 years old might have given birth to more than two children. These two associations are supported by study findings from Jigjiga, Ethiopia, where younger mothers and those who gave birth at a health facility had more odds of vaccinating their child [20].

## Limitations

Because mothers’ responses were believed to identify whether the child is vaccinated or not, this might result in recall and response biases.

The small sample size of this study might have drawbacks on the statistical test of checking association between the variables.

Inadequate similar literatures also limited the ability of this research to have an adequate discussion.

Because assessing vaccination status for the street children is the issue of measuring inequality of opportunity, downward bias or upward bias can be considered as another limitation for this study. Both biases may lead to a substantial underestimation or overestimation of the real level of inequality of opportunity.

## Conclusion

The magnitude of non-vaccinated children in this study is an attention-seeking figure. Street mothers younger than 20 years and those who had a home birth have lower odds of vaccinating their children than their counterparts.

## Data Accessibility Statement

All data generated or analyzed during this study are included in this published article.

## Abbreviations

BCG – Bacillie Calmiette Guerrie

DPT – Diptheria, Pertussis and Tetanus

DHS – Demographic and Health survey

EDHS – Ethiopian Demographic and Health survey

OPV – Oral Polio Vaccine

VPDs – Vaccine preventable diseases

WHO – World Health Organization
